# Scleroderma-like Impairment in the Network of Telocytes/CD34^+^ Stromal Cells in the Experimental Mouse Model of Bleomycin-Induced Dermal Fibrosis

**DOI:** 10.3390/ijms222212407

**Published:** 2021-11-17

**Authors:** Irene Rosa, Eloisa Romano, Bianca Saveria Fioretto, Daniele Guasti, Lidia Ibba-Manneschi, Marco Matucci-Cerinic, Mirko Manetti

**Affiliations:** 1 Department of Experimental and Clinical Medicine, Section of Anatomy and Histology, University of Florence, 50134 Florence, Italy; irene.rosa@unifi.it (I.R.); daniele.guasti@unifi.it (D.G.); lidia.ibba@unifi.it (L.I.-M.); 2 Department of Experimental and Clinical Medicine, Division of Rheumatology, University of Florence, 50134 Florence, Italy; eloisa.romano@unifi.it (E.R.); biancafioretto@icloud.com (B.S.F.); marco.matuccicerinic@unifi.it (M.M.-C.)

**Keywords:** dermal fibrosis, mouse model, scleroderma, skin, systemic sclerosis, telocytes/CD34^+^ stromal cells

## Abstract

Considerable evidence accumulated over the past decade supports that telocytes (TCs)/CD34^+^ stromal cells represent an exclusive type of interstitial cells identifiable by transmission electron microscopy (TEM) or immunohistochemistry in various organs of the human body, including the skin. By means of their characteristic cellular extensions (telopodes), dermal TCs are arranged in networks intermingled with a multitude of neighboring cells and, hence, they are thought to contribute to skin homeostasis through both intercellular contacts and releasing extracellular vesicles. In this context, fibrotic skin lesions from patients with systemic sclerosis (SSc, scleroderma) appear to be characterized by a disruption of the dermal network of TCs, which has been ascribed to either cell degenerative processes or possible transformation into profibrotic myofibroblasts. In the present study, we utilized the well-established mouse model of bleomycin-induced scleroderma to gain further insights into the TC alterations found in cutaneous fibrosis. CD34 immunofluorescence revealed a severe impairment in the dermal network of TCs/CD34^+^ stromal cells in bleomycin-treated mice. CD31/CD34 double immunofluorescence confirmed that CD31^−^/CD34^+^ TC counts were greatly reduced in the skin of bleomycin-treated mice compared with control mice. Ultrastructural signs of TC injury were detected in the skin of bleomycin-treated mice by TEM. The analyses of skin samples from mice treated with bleomycin for different times by either TEM or double immunostaining and immunoblotting for the CD34/α-SMA antigens collectively suggested that, although a few TCs may transition to α-SMA^+^ myofibroblasts in the early disease stage, most of these cells rather undergo degeneration, and then are lost. Taken together, our data demonstrate that TC changes in the skin of bleomycin-treated mice mimic very closely those observed in human SSc skin, which makes this experimental model a suitable tool to (i) unravel the pathological mechanisms underlying TC damage and (ii) clarify the possible contribution of the TC loss to the development/progression of dermal fibrosis. In perspective, these findings may have important implications in the field of skin regenerative medicine.

## 1. Introduction

Growing evidence indicates that dermal tissue structuring and remodeling during skin development and repair depends on the existence of different fibroblast populations [1,2]. For instance, it has been shown that embryonic skin fibroblasts originate from two distinct lineages, one forming the upper dermis including the hair growth-regulating dermal papilla and the arrector pili muscle, while the other giving rise to the reticular fibroblasts that synthesize the bulk of the fibrillar extracellular matrix of the lower dermis, and to the adipocytes and the stromal fraction that constitute the dermal white adipose tissue [1,2].

In such a scenario, the relatively recent discovery of telocytes (TCs) as a distinctive cell type populating the stromal compartment of the skin and other organs added further complexity to fibroblast heterogeneity [3,4,5,6,7,8]. Indeed, a significant body of evidence accumulated over the last decade indicates that TCs are ultrastructurally and immunophenotypically different from ‘classical’ fibroblasts [3,4,8,9,10]. In particular, TCs are distinguishable by transmission electron microscopy (TEM) as stromal cells with telopodes, which are extremely long cytoplasmic processes with a moniliform profile due to the alternation of very slender segments (podomers) and small dilated portions (podoms) [3,4,11,12]. When instead studying TCs by light microscopy using immunohistochemical techniques, it must be kept in mind that currently TC-specific antigens have yet to be identified, and that their antigenic profiles can vary according to the tissue/organ considered [4,6,7,13]. Nevertheless, at almost every anatomical site in which they have been identified, TCs appear to express CD34, which led to the widely accepted definition of TCs/CD34^+^ stromal cells [6,8,10,14]. In general, TCs/CD34^+^ stromal cells are spatially arranged with their telopodes forming a scaffold-like interstitial network, which is thought to provide support to neighboring cells through both intercellular contacts and transfer of extracellular vesicles carrying molecular signals as various as cytokines, growth factors, mRNAs, and epigenetic regulators such as miRNAs and other non-coding RNAs [3,4,7,15]. Extensive attention has also been paid to the unique relationship of TCs with tissue-resident stem/progenitor cells within specialized niches of different organs [6]. Hence, the likelihood that TCs may behave as a kind of guide for stem cells or function themselves as progenitors capable of differentiating into other cell types has raised a lot of expectations about their possible use in the field of regenerative medicine [6,16,17,18]. Concerning the origin of TCs/CD34^+^ stromal cells, they may derive in the embryo from mesenchymal stem cells (mesodermal origin) or from the neural crest, depending on location, while in postnatal life their source remains mostly unclear [8].

In the skin in physiological conditions, TCs/CD34^+^ stromal cells form an extensive scaffold-like cellular network which compartmentalizes the dermis, where telopodes intimately surround microvessels, nerve endings, and skin adnexa, and establish intercellular contacts with a variety of cell types, such as fibroblasts, macrophages, mast cells, and tissue-resident stem cells [9,19,20,21,22,23]. Owing to their peculiar spatial distribution and multitude of cell-to-cell communications together with an extraordinary ability to release different kinds of extracellular vesicles, TCs are regarded as important players in the intercellular signaling mechanisms underlying local tissue homeostasis, whose disruption may result into different disorders [4,6,7,14,24,25,26,27,28]. Thus far, abnormalities in TCs/CD34^+^ stromal cells have been reported in a variety of skin pathologies, ranging from tumor and tumor-like conditions to inflammatory and fibrosing/sclerosing diseases such as systemic sclerosis (SSc, scleroderma), a complex connective tissue disease in which immune system disturbances and microvascular injury evolve into progressive generalized fibrosis of the skin and internal organs [23,28,29,30,31,32,33,34,35,36]. In this regard, by means of TEM and CD34 immunostaining, fibrotic skin lesions from patients with SSc were found to be characterized by a severe impairment in the dermal network of TCs/CD34^+^ stromal cells, but it remains to be clarified in which extent this may be ascribed to either cellular degeneration and loss or possible transformation into disease-triggering profibrotic myofibroblasts [23,31,32].

In order to gain further insights into the implications of TCs/CD34^+^ stromal cells in skin fibrogenesis, we considered essential studying these cells in a well-established experimental model of the human disease. Therefore, the aim of the present work was to investigate whether the alterations of dermal TCs/CD34^+^ stromal cells found in human SSc [31] could be reproduced in a widely used model in which daily subcutaneous injections of the antitumor antibiotic bleomycin result into the development of localized dermal fibrosis that mimics several key features of SSc skin pathology [37,38].

## 2. Results

### 2.1. Verification of the Effective Development of the Bleomycin-Induced Dermal Fibrosis Mouse Model

**Figure 1:**
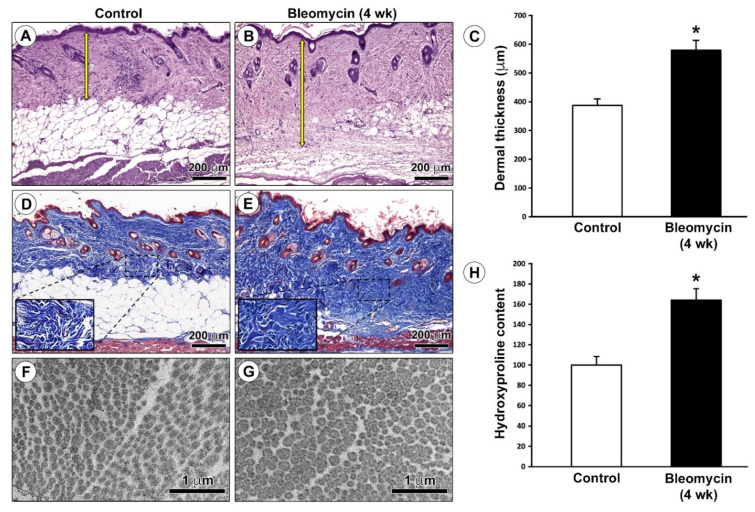
Verification of the mouse model of bleomycin-induced dermal fibrosis. (**A**,**B**) Representative photomicrographs of hematoxylin- and eosin-stained skin sections from (**A**) control mice and (**B**) mice treated with subcutaneous injections of bleomycin for 4 weeks. Yellow double head arrows indicate the distance between the dermal–epidermal junction and the dermal fibrous connective tissue–dermal white adipose tissue junction (dermal thickness). Scale bar: 200 μm (**A**,**B**). (**C**) Quantification of dermal thickness (μm). Columns represent the mean and whiskers represent the standard error of the mean; *n* = 6 mice in each group. * *p* < 0.001 vs. control (unpaired Student’s *t*-test). (**D**,**E**) Representative photomicrographs of skin sections from (**D**) control mice and (**E**) bleomycin-treated mice stained with Masson’s trichrome. The extracellular matrix is stained blue, while cytoplasm is stained red. Note the accumulation of extracellular matrix that replaces dermal white adipose tissue in the skin of bleomycin-treated mice. Insets in (**D**,**E**) represent higher magnifications of the boxed areas showing more abundant collagen fibers in the dermis of bleomycin-treated mice compared with control mice. Scale bar: 200 μm (**D**,**E**). (**F**,**G**) Representative transmission electron microscopy photomicrographs of skin ultrathin sections from (**F**) control mice and (**G**) bleomycin-treated mice stained with UranyLess and alkaline bismuth subnitrate solutions. Cross-sectioned dermal collagen fibrils of bleomycin-treated mice appear thickened and more closely packed than in control mice. Scale bar: 1 μm (**F**,**G**). (**H**) Quantification of hydroxyproline content expressed as a percent of the observed in control mice. Columns represent the mean, and whiskers represent the standard error of the mean; *n* = 6 mice in each group. * *p* < 0.001 vs. control (unpaired Student’s *t*-test). wk, weeks.

We first verified the successful establishment of our experimental model of dermal fibrosis by assessing general cutaneous histological features and measuring dermal thickness and collagen content in skin samples excised from mice treated with subcutaneous injections of bleomycin or saline (control) for 4 weeks. Histological examination of hematoxylin- and eosin-stained tissue sections demonstrated the presence of skin sclerotic changes with a considerable increase in dermal thickness of bleomycin-treated mice as compared with age- and sex-matched control mice (Figure 1A,B). Indeed, quantitative analysis revealed that dermal thickness, defined as the distance between the dermal–epidermal junction and the dermal fibrous connective tissue–dermal white adipose tissue junction, was significantly greater in the skin of bleomycin-injected mice than in that of control mice (*p* < 0.001; Figure 1C). Moreover, Masson’s trichrome staining showed a substantial accumulation of extracellular matrix with more abundant collagen fibers replacing dermal white adipose tissue in the skin of bleomycin-treated mice with respect to control mice (Figure 1D,E). Consistent with these light microscopy findings, cross-sectioned dermal collagen fibrils of bleomycin-treated mice appeared thickened and more closely packed than in control mice when analyzed by TEM (Figure 1F,G). In addition, we carried out colorimetric quantification of hydroxyproline for determination of dermal collagen content in murine skin samples. As shown in Figure 1H, the hydroxyproline content was significantly increased in the skin of bleomycin-treated mice compared with control mice (*p* < 0.001).

### 2.2. Impairment in the Network of Telocytes/CD34^+^ Stromal Cells in the Dermis of Bleomycin-Treated Mice

**Figure 2:**
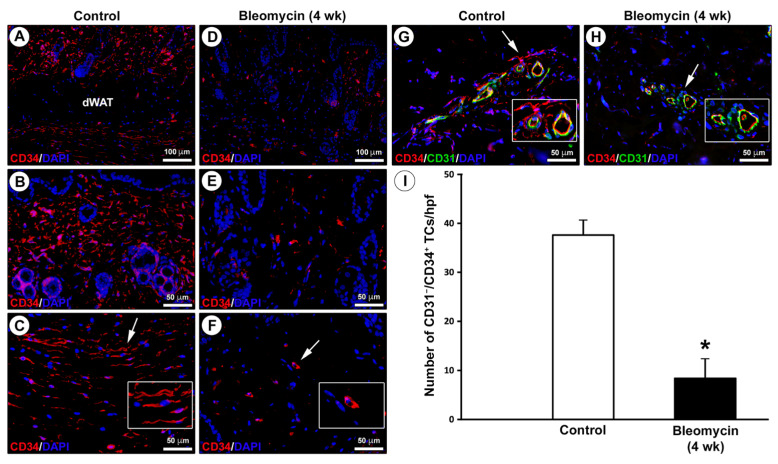
Severe impairment in the dermal network of telocytes (TCs)/CD34^+^ stromal cells in bleomycin-treated mice. (**A**–**F**) Representative photomicrographs of skin sections from (**A**–**C**) control mice and (**D**–**F**) mice treated with subcutaneous injections of bleomycin for 4 weeks subjected to immunofluorescence staining for CD34 (red) and 4′,6-diamidino-2-phenylindole (DAPI; blue) counterstain for nuclei. Insets in (**C**,**F**) represent higher magnifications of the tissue areas pointed by arrows. In control mouse skin, CD34^+^ stromal cells displaying a spindle-shaped morphology with long cytoplasmic processes and, therefore, identifiable as TCs, form a network that extends in the entire dermis, apart from dermal white adipose tissue (dWAT), and surrounds cutaneous adnexa (**A**–**C**). A few CD34^+^ stromal cells with no obvious cytoplasmic extensions are scattered in the dermis of bleomycin-treated mice (**D**–**F**). Scale bar: 100 μm (**A**,**D**), 50 μm (**B**,**C**,**E**,**F**). (**G**,**H**) Representative photomicrographs of skin sections from (**G**) control mice and (**H**) bleomycin-treated mice double immunostained for CD34 (red) and CD31 (green) with DAPI (blue) counterstain. Insets in (**G**,**H**) represent higher magnifications of the dermal tissue areas pointed by arrows. In control mouse dermis, TCs are identifiable as CD31^−^/CD34^+^ stromal cells; endothelial cells of blood microvessels are CD31^+^/CD34^+^ (**G**). In the dermis of bleomycin-treated mice, CD31^−^/CD34^+^ TCs are very few or even almost undetectable (**H**). Scale bar: 50 μm (**G**,**H**). (**I**) Quantitative analysis of CD31^−^/CD34^+^ TCs per high-power field (hpf). Columns represent the mean, and whiskers represent the standard error of the mean; *n* = 6 mice in each group. * *p* < 0.001 vs. control (unpaired Student’s *t*-test). wk, weeks.

In keeping with previous studies on human skin [23,31], the distribution of TCs/CD34^+^ stromal cells in skin sections from mice treated with subcutaneous injections of bleomycin or saline (control) for 4 weeks was firstly investigated by CD34 immunostaining (Figure 2A–F). In control mouse skin, CD34^+^ stromal cells displaying a spindle-shaped morphology with long cytoplasmic extensions and, therefore, identifiable as TCs (i.e., TCs/CD34^+^ stromal cells), were arranged in a widespread network extending in the entire dermis, except for dermal white adipose tissue, and surrounding cutaneous adnexa (Figure 2A–C). Conversely, a few CD34^+^ stromal cells with a roundish/oval shape and no obvious cytoplasmic extensions were detected in the fibrotic dermis of bleomycin-treated mice (Figure 2D–F).

Since CD34 is also expressed by endothelial cells of blood microvessels that may be misidentified as spindle-shaped TCs when sectioned tangentially with no obvious lumen, we further analyzed mouse skin sections by double immunofluorescence for CD34 and the endothelial marker CD31, which is commonly employed to distinguish accurately between CD31^−^/CD34^+^ TCs and CD31^+^/CD34^+^ microvessels (Figure 2G,H) [6,31,39,40]. This analysis confirmed the presence of a dermal network of CD31^−^/CD34^+^ TCs surrounding CD31^+^/CD34^+^ blood microvessels in control mouse skin, while CD31^−^/CD34^+^ TCs were very few or even almost undetectable in the dermis of bleomycin-treated mice (Figure 2G,H). As displayed in Figure 2I, quantitative analysis revealed that the number of CD31^−^/CD34^+^ TCs was significantly reduced in the skin of bleomycin-treated mice compared with control mice (*p* < 0.001).

**Figure 3:**
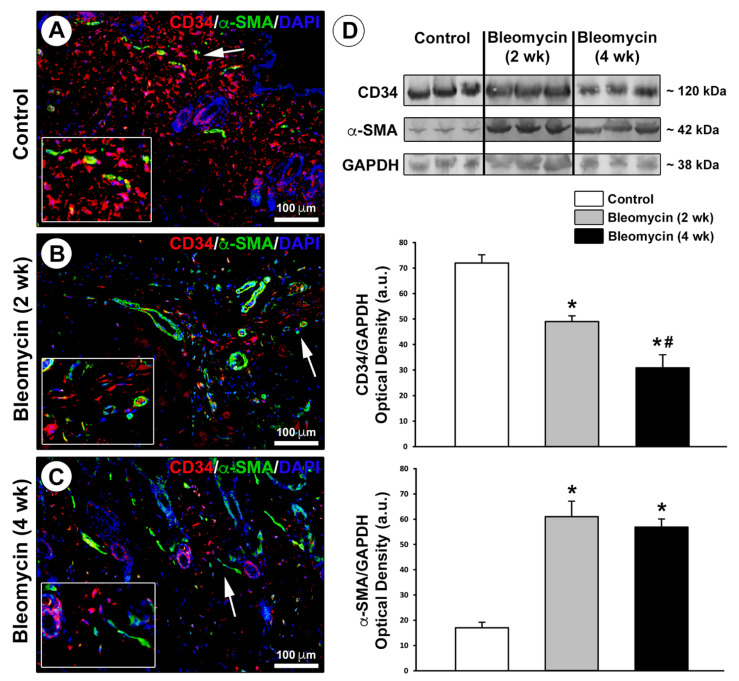
Changes in the expression of CD34 and α-smooth muscle actin (α-SMA) in the skin of bleomycin-treated mice. (**A**–**C**) Representative photomicrographs of skin sections from (**A**) control mice and (**B**,**C**) mice treated with subcutaneous injections of bleomycin for 2 weeks (early disease stage) or 4 weeks (sclerotic skin) subjected to double immunofluorescence staining for CD34 (red) and α-SMA (green) and 4′,6-diamidino-2-phenylindole (DAPI; blue) counterstain for nuclei. Insets in (**A**–**C**) represent higher magnifications of the dermal tissue areas pointed by arrows. In control mouse skin, telocytes (TCs)/CD34^+^ stromal cells form a widespread dermal network and do not express α-SMA, which is detectable only in microvascular structures (**A**). The dermis of mice treated with bleomycin for 2 or 4 weeks is characterized by a substantial reduction in TCs/CD34^+^ stromal cells along with the presence of some α-SMA^+^ myofibroblasts; a few stromal cells co-expressing CD34 and α-SMA are observed only in the skin of mice treated with bleomycin for 2 weeks (**B**,**C**). Scale bar: 100 μm (**A**–**C**). (**D**) Western blotting analysis of CD34 and α-SMA protein expression in skin lysates from control mice and mice treated with subcutaneous injections of bleomycin for 2 or 4 weeks. Representative immunoblots are shown at the top. Glyceraldehyde 3-phosphate dehydrogenase (GAPDH) was measured as a loading control. Molecular weight values (kDa) are indicated on the right. Results of densitometric analysis of the bands are shown at the bottom. Columns represent the mean, and whiskers represent the standard error of the mean; *n* = 6 mice in each group. * *p* < 0.001 vs. control, ^#^ *p* < 0.01 vs. bleomycin (2 wk) (Tukey’s test). wk, weeks.

To investigate whether TCs/CD34^+^ stromal cells could transdifferentiate into myofibroblasts by losing the CD34 marker and acquiring the expression of α-smooth muscle actin (α-SMA) as proposed in several pathologies including tissue fibrosis [16,23,41], skin sections from control and bleomycin-treated mice were subjected to CD34/α-SMA double immunostaining (Figure 3A–C). In addition to skin specimens from mice treated with bleomycin for 4 weeks that displayed sclerotic changes, we considered it was important to include in this analysis also skin specimens from mice injected with bleomycin for only 2 weeks as an early disease stage group. In control mouse dermis, the extensive network of TCs/CD34^+^ stromal cells did not express α-SMA, which was detectable only in pericytes/smooth muscle cells residing in the wall of microvessels (Figure 3A). The dermis of mice treated with bleomycin for 2 or 4 weeks instead displayed an impressive reduction in TCs/CD34^+^ stromal cells along with the presence of some α-SMA^+^ myofibroblasts (Figure 3B,C). A few stromal cells co-expressing CD34 and α-SMA, which were presumably TCs/CD34^+^ stromal cells transitioning to α-SMA^+^ myofibroblasts, could be observed in the skin of mice administered with bleomycin for 2 weeks, but not in that of mice treated for 4 weeks (Figure 3B,C).

Next, we quantified changes in CD34 and α-SMA protein expression levels by western blotting analysis on skin lysates from control mice and bleomycin-treated mice (Figure 3D). CD34 protein expression was significantly reduced after 2 weeks of bleomycin treatment (*p* < 0.001 vs. control mice), which was followed by a further significant reduction in CD34 protein levels after 4 weeks of treatment (*p* < 0.01 vs. mice treated with bleomycin for 2 weeks; Figure 3D). As far as α-SMA is concerned, α-SMA protein levels were significantly increased after 2 weeks of bleomycin treatment (*p* < 0.001 vs. control mice), without any further significant rise in protein expression after 4 weeks of treatment (Figure 3D). Thus, the progressive reduction in CD34 expression was not paralleled by a progressive increase in α-SMA expression during the development of bleomycin-induced dermal fibrosis (Figure 3D).

**Figure 4:**
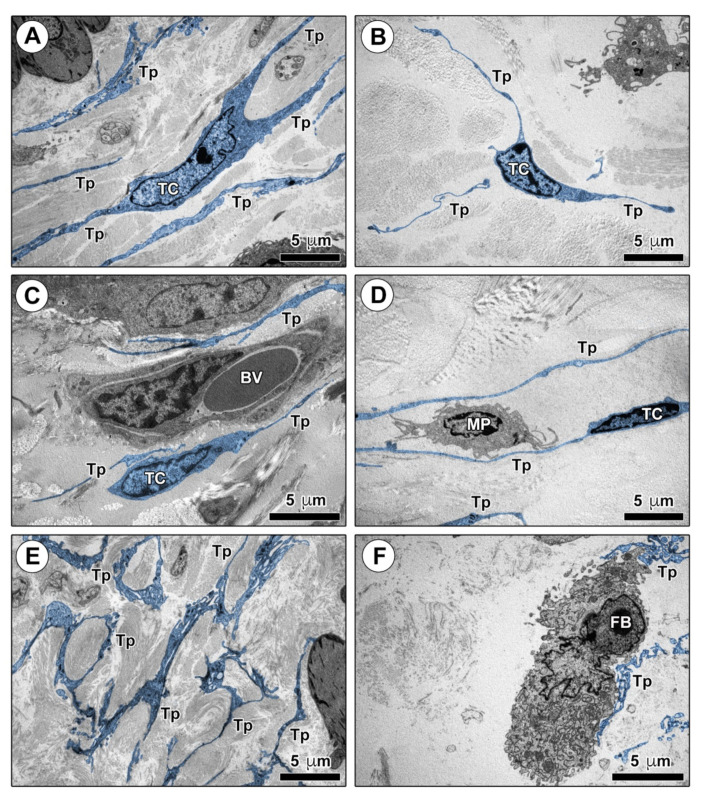
Ultrastructural identification of telocytes (TCs) in control mouse skin. (**A**–**F**) Representative transmission electron microscopy photomicrographs of skin ultrathin sections from control mice stained with UranyLess and bismuth subnitrate solutions. TCs and telopodes have been digitally colored in blue. TCs are ultrastructurally identifiable as stromal cells characterized by (i) a spindle-shaped, oval, or piriform cell body with a relatively large euchromatic nucleus surrounded by a scarce cytoplasm, and (ii) the presence of telopodes, long cytoplasmic processes with a narrow emergence from the cell body and a moniliform appearance due to the alternation of thin segments (podomers) and expanded portions (podoms). TCs are widely distributed in control mouse dermis, where their long telopodes surround collagen bundles (**A**,**B**) and blood microvessels (**C**), and establish intercellular contacts with macrophages (**D**). Telopodes often form a labyrinth-like network distributed among dermal collagen bundles (**E**) and contact fibroblasts (**F**). Scale bar: 5 μm (**A**–**F**). BV, blood vessel; FB, fibroblast; MP, macrophage; TC, telocyte; Tp, telopode.

**Figure 5:**
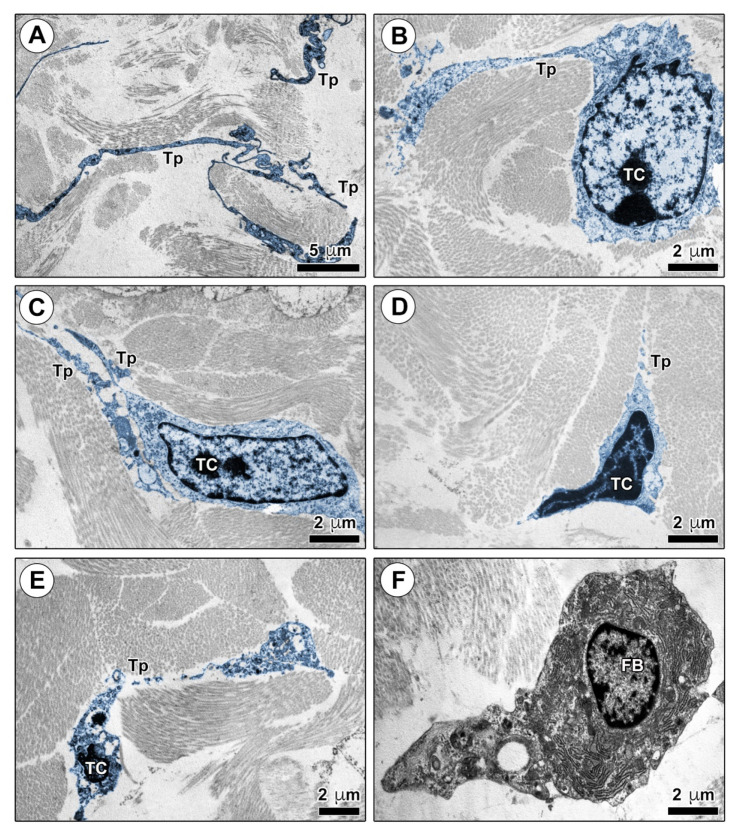
Ultrastructural alterations of telocytes (TCs) in the skin of bleomycin-treated mice. (**A**–**F**) Representative transmission electron microscopy photomicrographs of skin ultrathin sections from mice treated with subcutaneous injections of bleomycin for 2 weeks (**A**–**C**) or 4 weeks (**D**–**F**) stained with UranyLess and bismuth subnitrate solutions. TCs and telopodes have been digitally colored in blue. The network of telopodes appears reduced in the dermis of mice treated with bleomycin for 2 weeks (**A**); TCs embedded in a matrix composed of thickened and closely packed collagen bundles often display swollen cell body and telopodes with prominent cytoplasmic vacuolization (**B**,**C**). Shrinkage, shortening and breaking of telopodes along with apoptotic chromatin condensation and nuclear fragmentation are evident in the few TCs detectable in the dermis of mice treated with bleomycin for 4 weeks (**D**,**E**); fibroblasts are not in contact with telopodes (**F**). Scale bar: 5 μm (**A**), 2 μm (**B**–**F**). FB, fibroblast; TC, telocyte; Tp, telopode.

Finally, we analyzed the ultrastructural morphological features of TCs in mouse skin ultrathin sections by TEM (Figure 4 and Figure 5). As shown in Figure 4A–D, numerous dermal cells with the distinctive ultrastructural traits of TCs were identifiable in control mouse skin. Murine dermal TCs displayed a spindle-shaped, oval, or piriform cell body with a relatively large euchromatic nucleus surrounded by a scarce cytoplasm, and very long and thin telopodes with a narrow emergence from the cell body and the characteristic moniliform silhouette conferred by the alternation of podomers and podoms (Figure 4A–D). Telopodes appeared widely distributed throughout the mouse dermis, where they often formed a labyrinth-like network among collagen bundles, closely surrounded the basal lamina of blood microvessels, and established intercellular contacts with macrophages and fibroblasts (Figure 4A–F).

TEM analysis both confirmed the reduction in TCs and revealed a number of ultrastructural signs of TC injury in the skin of bleomycin-treated mice (Figure 5A–E). A substantial reduction in the labyrinth-like network of telopodes was already evident in the dermis of mice treated with bleomycin for 2 weeks as compared with control mice (Figure 4E and Figure 5A). After 2 weeks of bleomycin administration, dermal TCs appeared embedded in a matrix composed of thickened and closely packed collagen bundles, and often displayed swollen cell body and telopodes with prominent cytoplasmic vacuolization (Figure 5B,C). As far as skin ultrathin sections from mice administered with bleomycin for 4 weeks are concerned, the few detectable dermal TCs exhibited evident signs of cellular degeneration as shrinkage, shortening, and breaking of telopodes, along with apoptotic chromatin condensation and nuclear fragmentation (Figure 5D,E). Of note, the findings of telopode shrinkage/shortening/breaking were in agreement with the results of CD34 immunofluorescence that disclosed the presence of a few CD34^+^ stromal cells with a roundish/oval shape and no obvious cytoplasmic processes in the sclerotic dermis of mice treated with bleomycin for 4 weeks (Figure 2F). At variance with control mouse skin, the majority of fibroblasts captured in skin ultrathin sections from bleomycin-treated mice did not appear in contact with telopodes (Figure 4F and Figure 5F).

## 3. Discussion

Since the introduction of TCs/CD34^+^ stromal cells in the scientific literature in 2010, numerous efforts were made to inspect their existence and decipher their morphofunctional features in a variety of tissues/organs [3,4,5,6,7,8,11,12,42,43]. As a result, a growing body of evidence indicates that this long neglected, but definitely distinct, stromal cell population may greatly contribute to the shaping of the local tissue microenvironment in both health and disease [4,6,7,8,12,25,26,27,28,43].

Although currently anomalies in TCs/CD34^+^ stromal cells have been detected in a number of human pathological tissues, much work has yet to be done to clarify their functional implications [14,23,25,26,27,28,29,30,31,44,45,46,47,48,49,50,51,52,53,54,55]. For instance, preclinical animal models reproducing tissue alterations of TCs/CD34^+^ stromal cells, much like those found in human diseases, may clearly represent an invaluable tool for elucidating the effective contribution of these cells to disease pathogenesis and/or pathophysiology, which could often only be supposed [25]. In this context, to our knowledge, this is the first study to investigate TCs/CD34^+^ stromal cells in the mouse model of bleomycin-induced dermal fibrosis, which is widely used in SSc research [37,38,56]. Indeed, we have previously shown that clinically involved skin of patients with SSc displays a progressive impairment in the dermal network of TCs/CD34^+^ stromal cells, starting from the early cutaneous disease stage up to almost their complete loss in the advanced stage [31,32]. Nevertheless, the question as to whether these findings may be primarily related to cellular degenerative processes, immunophenotypical changes such as loss of CD34 and acquisition of the myofibroblast marker α-SMA, or both, has not yet been fully elucidated [23,31,32]. Several animal models of SSc are available; however, some models display dermal inflammation followed by fibrosis resembling early disease stages, while some others mainly mimic autonomous fibroblast activation in more advanced disease [56,57]. Thus, in an attempt to gain further insights into the pathological mechanisms underlying the changes in TCs/CD34^+^ stromal cells that accompany the development of skin fibrosis, here, we have chosen to utilize the bleomycin-induced model, which mimics early stages of human SSc [37,56].

As the principal finding, we demonstrate for the first time that both the immunohistochemical and ultrastructural abnormalities in TCs/CD34^+^ stromal cells previously described in lesional skin of SSc patients [31] are faithfully reproduced in the skin of bleomycin-treated mice. Indeed, CD34 immunofluorescence revealed that the dermal network of TCs/CD34^+^ stromal cells is severely compromised in the skin of bleomycin-treated mice compared with control mice. Moreover, several ultrastructural signs of TC injury and degeneration have been detected in the skin of bleomycin-treated mice by TEM. Of note, the findings of our analyses of skin samples from mice treated with bleomycin for different times (i.e., 2 and 4 weeks of treatment) by either TEM or double immunofluorescence and immunoblotting for the CD34/α-SMA antigens collectively suggest that, although a few TCs may escape cell injury and transdifferentiate into α-SMA^+^ myofibroblasts in the early disease stage, most of these cells rather undergo degeneration, and then are progressively lost as fibrogenesis advances. In fact, ultrastructural evidence of TC damage was already present after 2 weeks of bleomycin administration. Furthermore, the progressive loss of CD34 was not accompanied by a parallel gain in α-SMA during the development of dermal fibrosis, thus suggesting that TC/CD34^+^ stromal cell-to-α-SMA^+^ myofibroblast transition might contribute only in part to the disappearance of TCs/CD34^+^ stromal cells in skin fibrogenesis. In the context of the above-discussed findings, we acknowledge that, due to the paucity of mouse tissue specimens, we could not carry out additional analyses (e.g., quantification of TCs as CD31^−^/CD34^+^ stromal cells and assessment of markers of cell death in the skin of mice treated with bleomycin for 2 weeks) to further strengthen our conclusions.

Since TCs are considered central regulators of the local microenvironment by communicating with many different cell types through both direct intercellular contacts and release of paracrine factors [4,6,7,32,43], a loss of the complex telopode meshwork would inevitably result in disrupted intercellular signaling and, consequently, impaired tissue homeostasis. In this regard, it is worth noting that, at variance with control mouse skin, usually fibroblasts did not appear in contact with the telopodes of TCs in the fibrotic skin of bleomycin-treated mice. Overall, the results obtained in the bleomycin-induced mouse model of skin fibrosis are in line with those previously reported in human SSc [31,32] and, hence, they suggest that the progressive reduction in the network of TCs/CD34^+^ stromal cells might contribute to dermal tissue remodeling by favoring an uncontrolled synthetic activity of fibroblasts and, possibly, their transition to profibrotic myofibroblasts. Besides fibroblasts, in the normal dermis, TCs establish contacts with tissue-resident macrophages, which are another cell type involved in the pathogenesis of tissue fibrosis [58]. In particular, alternatively activated (M2) macrophages have been proposed to be important inducers of wound healing and tissue fibrosis/remodeling in various fibrosing disorders, including SSc [59]. Interestingly, there is evidence that TCs may regulate macrophage activation/plasticity and enhance the differentiation of classically activated (M1) macrophages, instead of M2 macrophages [60,61]. Thus, it is tempting to speculate that the loss of TCs found in the skin of SSc patients and bleomycin-treated mice might even favor the polarization of dermal macrophages into profibrotic M2 macrophages, further contributing to perturbation of dermal tissue homeostasis. In addition, TCs are often located in close vicinity of microvessels and can regulate angiogenesis by secreting factors acting on endothelial cells [62,63]. Therefore, the progressive disappearance of TCs might also contribute to capillary rarefaction and disturbed angiogenesis, which are other hallmarks of SSc skin pathology [64].

Owing to the mostly descriptive nature of the analyses we carried out, the present study has some intrinsic limitations as it could not fully provide relevant mechanistic insights. Therefore, we believe that further in vivo studies employing the bleomycin-induced mouse model, as well as in vitro studies of TCs in co-culture with other dermal cell types, could help elucidating how TCs may modulate the phenotype of neighboring cells and, hence, the pathogenetic implications of their loss in skin fibrosis. In perspective, following the encouraging results obtained in experimental models of unilateral ureter obstruction-induced renal fibrosis and myocardial infarction [65,66], the bleomycin-induced mouse model of SSc may also represent an ideal system for preclinical testing whether TC transplantation might attenuate skin fibrosis. Of note, the recent development of a protocol for the selective purification and establishment of primary cultures of dermal TCs [10] supports the feasibility of such future experimental directions. Moreover, further in-depth investigations utilizing this mouse model will hopefully help identifying the pathogenetic mechanisms underlying the damage of TCs, which could even prove useful as novel therapeutic targets to slow down or halt the progression of skin fibrosis. Finally, since the subcutaneous bleomycin mouse model is widely used in the development of novel therapies for SSc [37,56], we believe that verifying if the efficacy of the tested therapeutic approaches associates with a regeneration of the dermal networks of TCs/CD34^+^ stromal cells might provide new valuable insights into this still enigmatic cellular entity of the skin microenvironment.

## 4. Materials and Methods

### 4.1. Mouse Model of Bleomycin-Induced Dermal Fibrosis

Dermal fibrosis was induced in 6-week-old pathogen-free male C57BL/6 mice (Charles River Laboratories, Calco, Lecco, Italy) by local injection of bleomycin. Mice received subcutaneous injections of 100 μL of bleomycin (West-Ward Pharmaceuticals, Eatontown, NJ, USA) dissolved in 0.9% NaCl (saline solution; Eurospital SpA, Trieste, Italy) at a concentration of 0.5 mg/mL into a defined shaved area of the upper back (1 cm^2^ size) every other day for 4 weeks (*n* = 6) to allow local development of skin sclerotic changes [67]. Mice subjected to subcutaneous injections of 0.9% NaCl (*n* = 6) served as controls. Another group of mice received subcutaneous injections of bleomycin only for 2 weeks (*n* = 6) to analyze dermal changes in an early disease stage. At the end of the experimental procedures, mice were anaesthetized intraperitoneally with chloral hydrate (400 mg/kg) and sacrificed by cervical dislocation. The injected skin was rapidly excised, divided in four pieces, and processed for histologic analysis (light microscopy and TEM), hydroxyproline assay, and western blotting.

Mice were housed in the Laboratory Animal Facility (CeSAL, Centro Stabulazione Animali da Laboratorio, University of Florence, Florence, Italy), maintained at 23 ± 1 °C with a 12 h light/dark cycle, fed with standard laboratory diet and tap water ad libitum. All of the animal handlings were carried out in agreement with the Directive 2010/63/EU of the European Parliament and of the European Union council (22 September 2010) on the protection of animals used for scientific purposes.

### 4.2. Histochemical Analysis and Evaluation of Dermal Thickness

Skin samples were spread onto a piece of filter paper and fixed in 10% buffered formalin, dehydrated in graded alcohol series, and embedded in paraffin. Tissue sections (5 μm thick) were cut using a Leica RM2255 rotary microtome (Leica Microsystems, Mannheim, Germany), deparaffinized in xylene and hydrated through graded alcohols to distilled water. For hematoxylin and eosin staining, skin sections were stained with Mayer’s hematoxylin (Sigma-Aldrich, St. Louis, MO, USA) for 15 min, rinsed in running tap water, counterstained with 1% Eosin Y aqueous solution (Bio-Optica, Milan, Italy) for 5 min, dehydrated through graded alcohols and cleared in xylene. Trichrome staining was carried out using the Masson’s trichrome with blue aniline staining kit (catalog no. 04-010802; Bio-Optica) according to the manufacturer’s protocol. Stained skin sections were observed under a Leica DM4000 B microscope (Leica Microsystems), and transmitted light images were acquired using a Leica DFC310 FX 1.4-megapixel digital color camera equipped with the Leica software application suite LAS V3.8 (Leica Microsystems).

For comparisons of dermal thickness, three hematoxylin and eosin-stained skin sections were examined from every mouse of both control and bleomycin-treated groups. Dermal thickness was calculated at ×10 microscopic magnification by measuring the distance between the dermal–epidermal junction and the dermal fibrous connective tissue–dermal white adipose tissue junction (μm) in five randomly selected fields for each skin section. Two different examiners (I.R. and M.M.) performed the evaluation blindly, and the result was the mean of the two different observations for each sample.

### 4.3. Hydroxyproline Assay

For determination of dermal collagen content, colorimetric quantification of hydroxyproline was performed in a small skin biopsy (3 mm diameter) taken from every animal, as described elsewhere [68]. Briefly, frozen skin tissues were dehydrated, weighed, and hydrolyzed in 6 N HCl at 120 °C for 3 h. After neutralization in 6 N NaOH, the samples were mixed with 0.06 M chloramine T and incubated for 20 min at room temperature. Next, 3.15 M perchloric acid and 20% *p*-dimethylaminobenzaldehyde were added, and samples were incubated for an additional 20 min at 60 °C. The absorbance was measured at 560 nm in duplicate with a microplate spectrophotometer.

### 4.4. Transmission Electron Microscopy

Small mouse skin specimens were fixed with 4% cacodylate-buffered glutaraldehyde solution (pH 7.4) for 2 h at room temperature, rinsed in a cacodylate-buffered solution supplemented with sucrose, post-fixed in 1% OsO4 (Electron Microscopy Sciences, Foster City, CA, USA) for 1 h at room temperature, dehydrated with graded alcohol series, immersed in propylene oxide, and embedded in Epon 812 resin (Sigma-Aldrich). Semithin sections (2 μm thick) were obtained with an RMC MT-X ultramicrotome (EMME3, Milan, Italy), stained with a toluidine blue solution in 0.1 M borate buffer, and observed under a light microscope to identify the dermal tissue areas suitable for TEM. Ultrathin sections (~70 nm thick) of the selected skin areas were cut with the same ultramicrotome using a diamond knife and stained sequentially with UranyLess (Electron Microscopy Sciences, Foster City, CA, USA) and alkaline bismuth subnitrate solutions. Ultrathin sections were examined and photographed under a JEOL JEM-1010 electron microscope (Jeol, Tokyo, Japan) equipped with a MegaView III high-resolution digital camera and imaging software (Jeol). TCs and telopodes detected in electron microscopy images were digitally colored in blue using Adobe Photoshop CS6 software (Adobe Systems, San Jose, CA, USA).

### 4.5. Fluorescence Immunohistochemistry

Paraffin-embedded mouse skin sections (5 μm thick) were deparaffinized, subjected to antigen unmasking in 10 mm sodium citrate buffer (pH 6.0; Sigma-Aldrich), incubated in 2 mg/mL glycine solution (Sigma-Aldrich) for 10 min to quench autofluorescence, and blocked for 1 h at room temperature with 1% bovine serum albumin (Sigma-Aldrich) in phosphate-buffered saline. Single immunofluorescence for CD34 and double immunofluorescence for CD34 and CD31 or α-SMA were performed by incubating tissue slides overnight at 4 °C with the following primary antibodies: rabbit monoclonal anti-CD34 (1:50; catalog no. ab81289; Abcam, Cambridge, UK), rat monoclonal anti-CD31 (1:100; catalog no. ab7388; Abcam), and mouse monoclonal anti-α-SMA (1:100; clone 1A4; catalog no. A5228; Sigma-Aldrich). For double immunolabeling, a mixture of rabbit and rat or mouse monoclonal antibodies was applied to tissue sections. The following goat secondary antibodies (45 min, room temperature) were employed to reveal primary ones: Rhodamine Red-X-conjugated anti-rabbit IgG (1:200; catalog no. R-6394; Invitrogen, San Diego, CA, USA), Alexa Fluor-488-conjugated anti-rat IgG (1:200; catalog no. A-11006; Invitrogen), and Alexa Fluor-488-conjugated anti-mouse IgG (1:200; catalog no. A-11001; Invitrogen). Negative controls were obtained by substituting primary antibodies with irrelevant isotype-matched and concentration-matched IgG (Sigma-Aldrich), while secondary antibodies cross reactivity was assessed by omitting primary antibodies. Nuclei were counterstained with 4′,6-diamidino-2-phenylindole (DAPI; Chemicon International, Temecula, CA, USA). The immunolabeled slides were mounted with an antifade aqueous mounting medium (Biomeda Gel Mount; Electron Microscopy Sciences) and examined under a Leica DM4000 B microscope (Leica Microsystems). Fluorescence images were captured with a Leica DFC310 FX 1.4-megapixel digital color camera (Leica Microsystems).

CD31^−^/CD34^+^ TCs were counted in six randomly chosen microscopic high-power fields (×40 magnification) per skin sample by two independent observers (I.R. and M.M.), who were blinded with regard to the sample classification. Only the cells with well-defined DAPI-stained nuclei were counted. The mean of the two different observations for each sample was used for statistical analysis.

### 4.6. Western Blotting

Proteins were extracted from mouse skin specimens by homogenization for 5 min in ice-cold lysis buffer [50 mM Tris HCl (pH 7.4), 150 mM NaCl, 1 mM EDTA, 1% Triton X-100, 0.25% sodium dodecyl sulfate] supplemented with 1 mM sodium orthovanadate, 1 mM NaF, 1 mM EDTA, 1 mM phenylmethylsulphonyl fluoride, and 10 µg/mL aprotinin. The solution was sonicated in an ultrasonic water bath, cleared by centrifugation, and assayed for protein content using Bradford’s method. For each sample, twenty micrograms of proteins were electrophoresed on NuPAGE 4 to 12% Bis-Tris Gel (Invitrogen) and blotted onto polyvinylidene difluoride membranes (Invitrogen). The membranes were blocked with blocking solution included in the Western Breeze Chromogenic Western Blot Immunodetection Kit (Invitrogen) for 30 min at room temperature on a rotary shaker and then incubated for 1 h at room temperature with rabbit anti-CD34 (1:1000; catalog no. ab81289; Abcam), rabbit anti-α-SMA (1:1000; catalog no. ab5694; Abcam), and mouse anti-glyceraldehyde 3-phosphate dehydrogenase (GAPDH; 1:5000; catalog no. ab8245; Abcam). Immunodetection was performed according to the Western Breeze Chromogenic Immunodetection protocol (Invitrogen). Densitometric analysis of the bands was carried out using ImageJ 1.49v software (NIH, Bethesda, MD, USA; http://rsbweb.nih.gov/ij, accessed on 8 March 2021). Each value was normalized to the respective GAPDH value.

### 4.7. Statistical Analysis

Statistical analysis was performed using the SPSS software for Windows, version 27.0 (Statistical Package for Social Sciences Inc., Chicago, IL, USA). Data are expressed as the mean ± standard error of the mean (SEM). After assessing the normality of data by Kolmogorov–Smirnov test, a one-way ANOVA with post-hoc Tukey’s test or unpaired Student’s *t*-test was used for statistical analyses, as appropriate. Values of *p* < 0.05 were considered statistically significant. Sample size was calculated with a priori power analysis (G*Power Version 3.1.9.2 for Windows; www.gpower.hhu.de, accessed on 12 May 2014), considering dermal thickening as endpoint. A sample size of six mice per group was determined sufficient to have a power > 0.80 at a significance level of *p* < 0.05.

## 5. Conclusions

In conclusion, our data demonstrate that TC/CD34^+^ stromal cell changes in the skin of bleomycin-treated mice mimic very closely those observed in human SSc skin [31], which makes this experimental model a suitable tool to (i) further dissect the pathological mechanisms underlying TC damage and (ii) clarify the possible contribution of the TC loss to the development/progression of dermal fibrosis. In perspective, these findings may have important implications in the field of skin regenerative medicine.

## Data Availability

All relevant data are included within the manuscript.
